# Extensive protein *S*-nitrosylation associated with human pancreatic ductal adenocarcinoma pathogenesis

**DOI:** 10.1038/s41419-019-2144-6

**Published:** 2019-12-04

**Authors:** Chaochao Tan, Yunfeng Li, Xiahe Huang, Meijin Wei, Ying Huang, Zhouqin Tang, He Huang, Wen Zhou, Yingchun Wang, Jiliang Hu

**Affiliations:** 1 Department of Clinical Laboratory, Hunan Provincial People’s Hospital, The First Affiliated Hospital of Hunan Normal University, Hunan Normal University, Changsha, 410005 China; 2 Clinical Laboratory of Translational Medicine Research Institute, Hunan Provincial People’s Hospital, The First Affiliated Hospital of Hunan Normal University, Hunan Normal University, Changsha, 410005 China; 3 Department of Hepatobiliary Surgery, Hunan Provincial People’s Hospital (The First Affiliated Hospital of Hunan Normal University), Changsha, 410005 China; 4 State Key Laboratory of Molecular Developmental Biology, Institute of Genetics and Developmental Biology, Chinese Academy of Sciences, Beijing, 100101 China; 5 School of Pharmaceutical Sciences, Guangzhou University of Chinese Medicine, Guangzhou, 510006 China; 6 Department of Emergency, Hunan Provincial People’s Hospital (The First Affiliated Hospital of Hunan Normal University), Changsha, 410005 China; 7 Department of Histology and Embryology, Xiangya School of Medicine, Central South University, Changsha, 410013 China; 8 Department of Histology and Embryology, School of Pre-clinical Medicine, Xinjiang Medical University, Urumqi, 830011 China

**Keywords:** Proteomics, Nitrosylation

## Abstract

NO (nitric oxide)-mediated protein *S*-nitrosylation has been established as one major signaling mechanism underlying cancer initiation and development, but its roles in PDAC (pancreatic ductal adenocarcinoma) pathogenesis still remain largely unexplored. In this study, we identified 585 unique *S-*nitrosylation sites among 434 proteins in PDAC patients and PANC-1 cell line by a site-specific proteomics. Larger number of *S-*nitrosylated proteins were identified in PDAC tissues and PANC-1 cells than adjacent non-cancerous tissues. These *S-*nitrosylated proteins are significantly enriched in a multitude of biological processes associated with tumorigenesis, including carbohydrate metabolism, cytoskeleton regulation, cell cycle, focal adhesion, adherent junctions, and cell migration. Components of the pancreatic cancer pathway were extensively *S-*nitrosylated, such as v-raf-1 murine leukemia viral oncogene homolog 1 (Raf-1) and Signal transducer and activator of transcription 3 (STAT3). Moreover, NOS (NO synthase) inhibitor significantly repressed STAT3 *S*-nitrosylation in PANC-1 cells, which caused significant increase of STAT3 phosphorylation and PANC-1 cell viability, suggesting important roles of protein *S*-nitrosylation in PDAC development. These results revealed extensive protein *S*-nitrosylation associated with PDAC pathogenesis, which provided a basis for protein modification-based cancer diagnosis and targeted therapy.

## Introduction

Pancreatic ductal adenocarcinoma (PDAC), originated from exocrine cells in pancreas, is the most common pancreatic cancer subtype accounting for over 85% of malignant cases in pancreas^1^. Globally, PDAC remains one of the major causes of cancer death, featured by poor prognosis and a five-year survival rate of less than 8%^2,3^. Although a panel of genetic mutations were established as driving forces for pancreatic adenocarcinoma development such as KRAS and P53^4^, their application as therapeutic targets were only effective in a small percentage of PDAC patients^2^. Alternative roads based on transcriptome analysis, stroma depletion, and protein post-translational modifications have recently been considered as potential strategies^1,2,5^, but need to be further investigated.

Nitric oxide (NO), produced by NO synthase (NOS), serves as a critical signaling molecule in tumorigenesis by regulating pleiotropic processes including cell proliferation, apoptosis, metastasis, angiogenesis, and chemoresistance^6,7^. In PDAC pathogenesis, dysregulated NO overproduction by inducible and endothelial (iNOS and eNOS) is critically involved in tumor development and associated with poor survival, but the underlying mechanism remains largely unexplored^8–11^. NO performs its biological functions mainly through protein *S-*nitrosylation, a redox modification during which a NO group is covalently added onto the cysteine (Cys) thiol of a protein to form *S-*nitrosothiol (SNO)^12,13^. *S-*nitrosylation exerts various molecular effects on protein activity, conformation, stability, subcellular distribution, and interaction networks^6,14–17^, thus involved in tumorigenesis and other processes^6,17–19^. A number of *S-*nitrosylated proteins were extensively investigated in cancer cells, such as B-cell lymphoma-2 (Bcl-2)^20^, Ras^21^, Fas^22^, mitogen-activated protein kinase phosphatase-1 (MKP-1)^23^, tumor necrosis factor receptor associated protein 1 (TRAP1)^24^ and Phosphatase and tensin homolog (PTEN)^25^. Particularly, *S-*nitrosylation of wild-type Ras protein is required for the initiation and maintenance of PDAC tumor growth^21^. Except for this, roles of protein *S-*nitrosylation in PDAC pathogenesis still remain poorly understood.

Mass spectrometry-based proteomics, combined with chemically selective approaches like biotin-switch and SNO trapping by triaryl phosphine, has made it feasible to identify protein *S-*nitrosylation in large scale^26–29^. *S-*nitrosoproteomics greatly facilitated the elucidation of protein *S-*nitrosylation underlying multiple processes in various species, including mitochondrial fatty acid metabolism, neural signaling, neurodegeneration, and Duchenne muscular dystrophy pathogenesis^30–33^. We previously identified large number of *S-*nitrosylation proteins induced by NO overproduction through site-specific proteomics^34^, which provided clues for functional investigations of *S-*nitrosylation signaling^35,36^. In this study, site-specific proteomic profiling of endogenously *S-*nitrosylated proteins in PDAC tissues and pancreatic cells were performed for novel insights into nitric oxide and protein *S*-nitrosylation during pancreatic cancer pathogenesis.

## Materials and methods

### Experimental design and statistical rationale

**Fig. 1:**
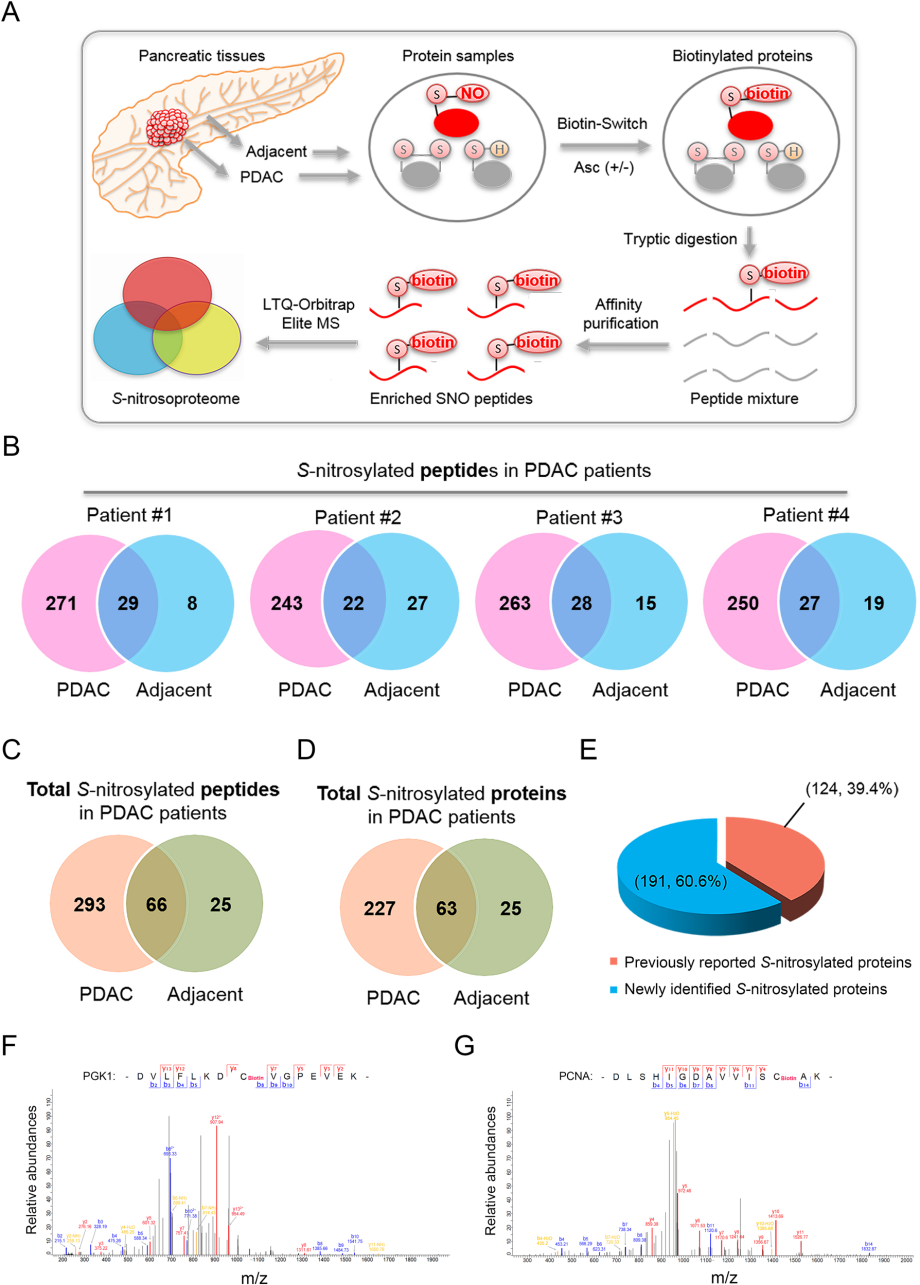
Site-specific identification of *S-*nitrosylated proteins in PDAC patients. **a** Schematic illustration of site-specific *S-*nitrosoproteomic analysis in pancreatic tissues. **b** *S-*nitrosylated peptides identified by site-specific proteomics in PDAC and adjacent tissues. Numbers of *S-*nitrosylated peptides in four PDAC patients were shown in Venn diagram. **c** Total numbers of *S-*nitrosylated peptides identified by site-specific proteomics in PDAC patients. Numbers of *S-*nitrosylated peptides were shown in a Venn diagram. **d** *S-*nitrosylated proteins identified by site-specific proteomics in pancreatic tissues. Proteins identified in both PDAC and adjacent tissues were shown in a Venn diagram. **e** Comparison of *S-*nitrosylated proteins identified between previous studies and this study. (**f**, **g**) Mass spectrometric identification of Cys-99 of PGK1 (**f**) and Cys-162 of PCNA (**g**) as *S-*nitrosylated residues by site-specific proteomics. SNO, *S-*nitrosylated proteins; PDAC pancreatic ductal adenocarcinoma, Asc sodium ascorbate, PGK1 phosphoglycerate kinase 1, PCNA proliferating cell nuclear antigen.

**Fig. 2:**
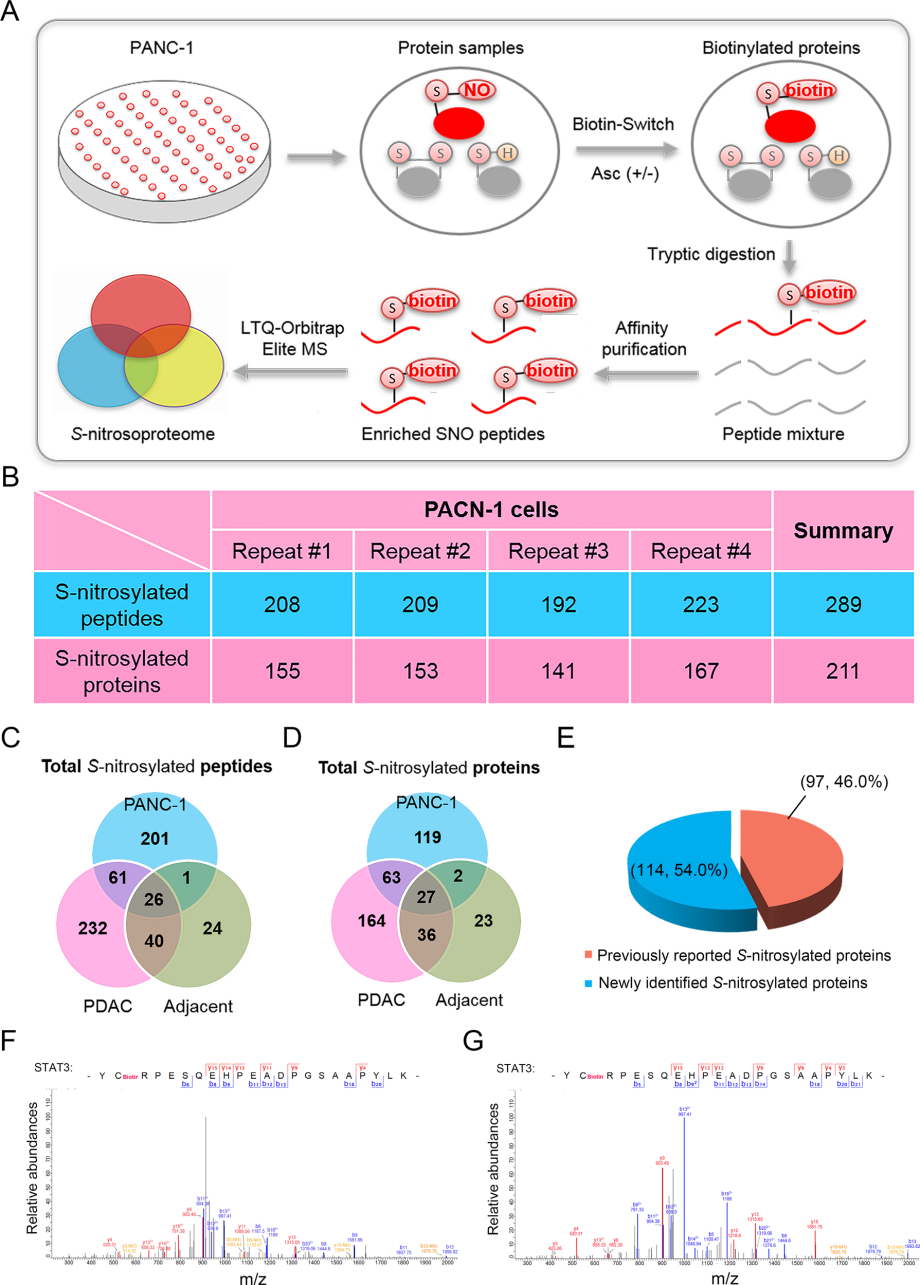
Site-specific identification of protein *S-*nitrosylation in PANC-1 cells. **a** Schematic illustration of *S-*nitrosoproteomic analysis using pancreatic cancer cells. **b** Numbers of *S-*nitrosylated peptides and proteins identified by four proteomic experiments in PANC-1 cells. **c** Total numbers of *S-*nitrosylated peptides in PANC-1 cells and pancreatic tissues shown in Vann diagram. **d** Total numbers of *S-*nitrosylated proteins in PANC-1 cells and pancreatic tissues shown in Vann diagram. **e** Comparison of *S-*nitrosylated proteins identified in PANC-1 cells with previous studies. (**f**, **g**) Mass spectrometric identification of Cys-687 of STAT3 protein in PDAC tissues (**f**) and PANC-1 cells (**g**) as *S-*nitrosylated residue. SNO *S-*nitrosylated proteins, PDAC pancreatic ductal adenocarcinoma, Asc sodium ascorbate, STAT3 signal transducers and activators of transcription 3.

Cancerous and adjacent non-cancerous pancreatic tissues collected from four patients with PDAC, as four biological replicates (*n* = 4), were separately analyzed by site-specific proteomics for discovery of *S-*nitrosylated proteins (See Fig. 1a for details). Limited by tissue volume collected from patients, each sample was analyzed by one biological replicate. Negative control without sodium ascorbate treatment during biotinylation of *S-*nitrosylated proteins was included for each cancerous or adjacent tissue, which were also analyzed by mass spectrometry to exclude false positive identification due to incomplete blocking. For identification of *S-*nitrosylated proteins in PANC-1 cells (see Fig. 2a for details), the site-specific proteomics were biologically repeated for four times and negative control (without sodium ascorbate treatment) was included in each replicate. Reliable identification of *S*-nitrosylated peptides were finally obtained by searching with MaxQuant (1.6.0.16) using a FDR of <1% at both the peptide and protein group levels for control of false identification.

### Tissue specimen

Pancreatic tissues specimens were collected from four early stage PDAC patients admitted into the Department of Digestive Surgery, Hunan Provincial People’s Hospital (Changsha, China). Detailed clinical data including clinical staging of PDAC patients were listed in Supplemental Table S1. Both the PDAC and paired adjacent non-cancerous pancreatic tissues were collected and freshly frozen in liquid nitrogen during surgical treatment. Histological evaluation was done by experienced pathologists through hematoxylin/eosin staining of tissue slides. The research was approved by the Ethical Committee of the Hunan Provincial People’s Hospital (Changsha, China) and carried out following the Declaration of Helsinki. Written informed consents were obtained from each subject.

### Cell culture and viability

PDAC cell lines PANC-1 and SW1990 were purchased from the Type Culture Collection of Chinese Academy of Sciences (Shanghai, China). The immortalized non-neoplastic human pancreatic ductal epithelium cell line HPDE6c7 was obtained from Kyushu University, Japan. Cells were authenticated by the short tandem repeat (STR) profiling method and cultured in Dulbecco’s Modified Eagle’s Medium (DMEM) containing 10% fetal bovine serum (Invitrogen) and penicillin and streptomycin at 37 °C. PANC-1 cell viability were determined by CCK-8 (Cell Counting Kit-8; Dojindo, Japan) following manufacturer’s instructions, after being treated with 10 μM L-NAME (N G-nitro-L-arginine methyl ester; Sigma-Aldrich) for 48 h.

### Biotinylation of *S-*nitrosylated proteins

Biotin-switch assay was performed to biotinylate endogenously *S-*nitrosylated proteins in pancreatic tissues or cells using *S-*alkylating labeling strategy and irreversible biotinylation procedure as previously described^26,27,34^. Briefly, approximately 0.8 g frozen specimens were made into fine powder in liquid nitrogen. Cultured cells were lyzed using cell lysis buffer containing 250 mM HEPES, 1% NP-40, 150 mM NaCl, 1 mM EDTA, 0.1 mM neocuproine, 1 mM PMSF and 1% protease inhibitor cocktail (Sigma-Aldrich). Tissue or cell lysates were then blocked by *S-*alkylation with 200 mM iodoacetamide at 37 °C for 1 h, and incubated with HENS buffer containing 1 mM sodium ascorbate and 4 mM biotin-maleimide (Sigma-Aldrich) at room temperature for 2 h in darkness. Negative control without sodium ascorbate treatment during biotinylation of *S-*nitrosylated proteins was included for each tissue or cell samples.

### Tryptic digestion and affinity purification

Proteomic identification of *S-*nitrosylated peptides in tissues and cells were carried out using site-specific method as previously described^34^. Briefly, biotinylated proteins were subjected to in-solution digestion with trypsin (Sigma-Aldrich) at 37 °C for 16 h, followed by affinity chromatography enrichment using NeutrAvidin agarose resins (Thermo Scientific). Purified peptides were resuspended in 0.1% FA, desalted using C18 Zip-tip (Millipore) and then used for mass spectrometric analysis.

### Liquid chromatography-mass spectrometric analysis

Mass spectrometric identification of biotinylated peptides was performed using a LTQ Orbitrap Elite mass spectrometer (Thermo Fisher Scientific) coupled online to an Easy-nLC 1000 (Thermo Fisher Scientific) in data-dependent mode. Peptides were first separated by reverse phase LC using 75 μm (ID) × 250 mm (length) analytical columns packed with C18 particles of 5 µm diameter. Mobile phases for liquid chromatography separation contain buffer A (0.1% FA) and buffer B (100% acetonitrile and 0.1% FA). Peptide separation was finished using a 90-min non-linear gradient (3–8% buffer B for 10 min, 8–20% buffer B for 1 h, 20–30% buffer B for 8 min, 30–100% buffer B for 2 min, and 100% buffer B for 10 min) with a flow rate of 300 nl/min. The MS measurements were carried out using the positive ion mode, and precursor ions were measured in the Orbitrap analyzer at 240,000 resolution (at 400 m/z) with a target value of 10^6^ ions. Twenty ions with the highest intensities from each MS scan were isolated, fragmented and measured in linear ion trap, and CID normalized collision energy was set to 35. The mass spectrometry proteomics data, including annotated spectrum images, have been deposited to the ProteomeXchange Consortium via the PRIDE^37^ partner repository with the dataset identifier PXD012512.

All raw MS files were collected and subjected to database search using the software MaxQuant (version 1.6.0.16). The UniProt proteome sequences containing 20244 entries including canonical and isoforms (taxonomy: Homo sapiens, released on 30/1/2018) were used for database searching. Trypsin was selected as the protease for protein digestion, and the maximum allowable miscleavages was set to two. The mass tolerances for precursor ions and fragments ions were set to 20 PPM. A minimum peptide length of six amino acids and a maximum peptide length of 144 amino acids were also applied for database search. Cysteine biotinylation (451.200 Da), cysteine carbamidomethylation, N-terminal acetylation and methionine oxidation were included as variable modification. A minimum score of 40 and false discovery rate (FDR) of 0.01 was used as the threshold for peptide and protein identifications. A localization probability of over 0.5 was employed as localization score threshold, and modified peptides with unambiguous site localization were listed in a separate supplemental table. Biotinylated peptides identified in corresponding negative controls (without sodium ascorbate treatment) were finally excluded from the *S-*nitrosylated peptide lists.

### Bioinformatics

Previously reported *S-*nitrosylated proteins were summarized by search against the SNObase database^38^, combined with recent publications. Consensus sequences of *S-*nitrosylated peptides were predicted using the pLogo method^39^. Gene ontology (GO) and KEGG pathway analysis were performed using the DAVID bioinformatics resource^40^. Pathway diagrams with enrichment of *S-*nitrosylated proteins were modified from the KEGG pathway database (www.kegg.jp).

### Immunoblotting

The immunoblotting assay was done as previously described^41^. Primary antibodies used in this study were listed as follows: anti-eNOS (ab199956; Abcam), anti-nNOS (ab5586; Abcam), anti-iNOS (ab15323; Abcam), anti-biotin (7075 S; Cell Signaling Technology), anti-Raf-1 (ab137435; Abcam), anti-STAT3 (ab109085; Abcam), anti-p-STAT3 (Y705) (ab76315; Abcam), and anti-β-actin antibodies (#5125; Cell Signaling Technology).

### Statistical analysis

Difference between groups were evaluated by the Student’s *t* test or ANOVA (analysis of variance) as appropriate using the SPSS 20.0 software. Significant differences were defined by a *P* value of <0.05.

## Results

### Site-specific identification of *S-*nitrosylated proteins in PDAC tissues

The expressions of three NOS proteins were first tested between PDAC and adjacent tissues. Abundances of three NO synthases iNOS, eNOS and nNOS (neuronal NOS) in PDAC tissues showed significant increase compared with paired adjacent tissues (Supplemental Fig. S1). We further found that the total *S-*nitrosylated protein (SNO) levels in pancreatic cancer tissues from PDAC patients were also significantly higher than corresponding adjacent tissues (Supplemental Fig. S2). Remarkable elevation of NOS expression and protein *S-*nitrosylation suggested that this NO-mediated protein modification might play central roles in PDAC pathogenesis. For a comprehensive view of protein *S-*nitrosylation, we used a site-specific proteomic approach to characterize *S-*nitrosylated proteins and modified Cys residues in pancreatic tissues collected from four PDAC patients (Supplemental Table S1). In this method, endogenously *S-*nitrosylated proteins in pancreatic tissues or cultured cells were first irreversibly biotinylated via biotin-switch, followed by tryptic digestion, biotin-affinity purification and final identification of protein identity and modification sites using LTQ Orbitrap Elite mass spectrometer. To improve the reliability, negative control without sodium ascorbate treatment during biotin-switch assay was included in analysis of each cancerous and adjacent tissues, which was also subjected to LC-MS/MS analysis^26,27,34^. Biotinylated peptides identified in negative controls were excluded from the corresponding *S-*nitrosylation dataset (Fig. 1a; Supplemental Table S2).

In pancreatic tissues collected from four PDAC patients, a total of 384 *S-*nitrosylated peptides were identified, consisting of 359 and 91 unique *S-*nitrosylated peptides in cancerous and adjacent tissues, respectively (Fig. 1b, c; Supplemental Tables S2–S4). These peptides were mapped to totally 315 *S-*nitrosylated proteins, containing 290 and 88 proteins endogenously *S-*nitrosylated in cancerous and adjacent tissues from PDAC patients (Fig. 1d; Supplemental Tables S3, S4). Peptides with ambiguous modification site assignments were listed in Supplemental Table S5. Significantly larger number of *S-*nitrosylated proteins identified in PDAC tissues, compared with paired adjacent tissues, is consistent with increased NO production and NOS expression shown in Supplemental Figs S1 and S2. Among these proteins, only 63 proteins were identified in both PDAC and adjacent tissues, which covers only 27.8% of *S-*nitrosylated proteins in PDAC tissues (Fig. 1d; Supplemental Table S3), showing remarkable differences of *S-*nitrosylation profiles between PDAC and adjacent tissues. Compared with previous studies in *Homo sapiens*, we found that 39.4% *S-*nitrosylated proteins (124/315) identified in our proteomic analysis were also previously reported, strongly validating the reliability of results obtained by this proteomic analysis (Fig. 1e and Supplemental Table S3). For instance, *S-*nitrosylated Cys residues were identified in Phosphoglycerate kinase 1 (PGK1) and proliferating cell nuclear antigen (PCNA), which are *S-*nitrosylated proteins reported by previous studies (Fig. 1f, g).

### Site-specific identification of *S-*nitrosylated proteins in PANC-1 cells

To get a more comprehensive SNO profile, we performed site-specific proteomic analysis of *S-*nitrosylated proteins in cultured PANC-1 cells with four biological repeats. Cell lysates without sodium ascorbate treatment were included as negative control (Fig. 2a; Supplemental Table S2). In PANC-1 cells, 289 unique *S-*nitrosylated peptides were identified by four biological repeats of site-specific proteomics, which were mapped to 211 *S-*nitrosylated proteins (Fig. 2b, c; Supplemental Tables S2, S6 and S7). Peptides with ambiguous modification site assignments were listed in Supplemental Table S8. Among these peptides identified in PANC-1 cells, 30.5% (88/289) were also identified in above-mentioned *S-*nitrosoproteomic analysis of pancreatic tissues (Fig. 2c; Supplemental Tables S6 and S7). Specifically, 87 *S-*nitrosylated peptides were identified in both the PDAC tissues and PANC-1 cells, which is much more than these 27 peptides identified in both the adjacent tissues and PANC-1 cells (Fig. 2c; Supplemental Table S6 and S7). Moreover, 42.7% (90/211) *S-*nitrosylated proteins in PANC-1 cells were also identified in PDAC tissues, while only 13.7% (29/211) *S-*nitrosylated proteins in PANC-1 cells were identified in adjacent non-cancerous pancreatic tissues (Fig. 2d; Supplemental Tables S6 and S7). In addition, we found that almost half (46.0%; 97/211) of *S-*nitrosylated proteins identified in PANC-1 cells were previously reported (Fig. 2e; Supplemental Tables S6 and S7), further confirming the reliability of our *S-*nitrosoproteomic data. For instance, the Cys-687 residue of Signal transducers and activators of transcription 3 (STAT3) was identified as *S-*nitrosylated site in both the PDAC tissues and PANC-1 cells (Fig. 2f, g).

### Consensus sequences of *S-*nitrosylated peptides in PDAC

**Fig. 3:**
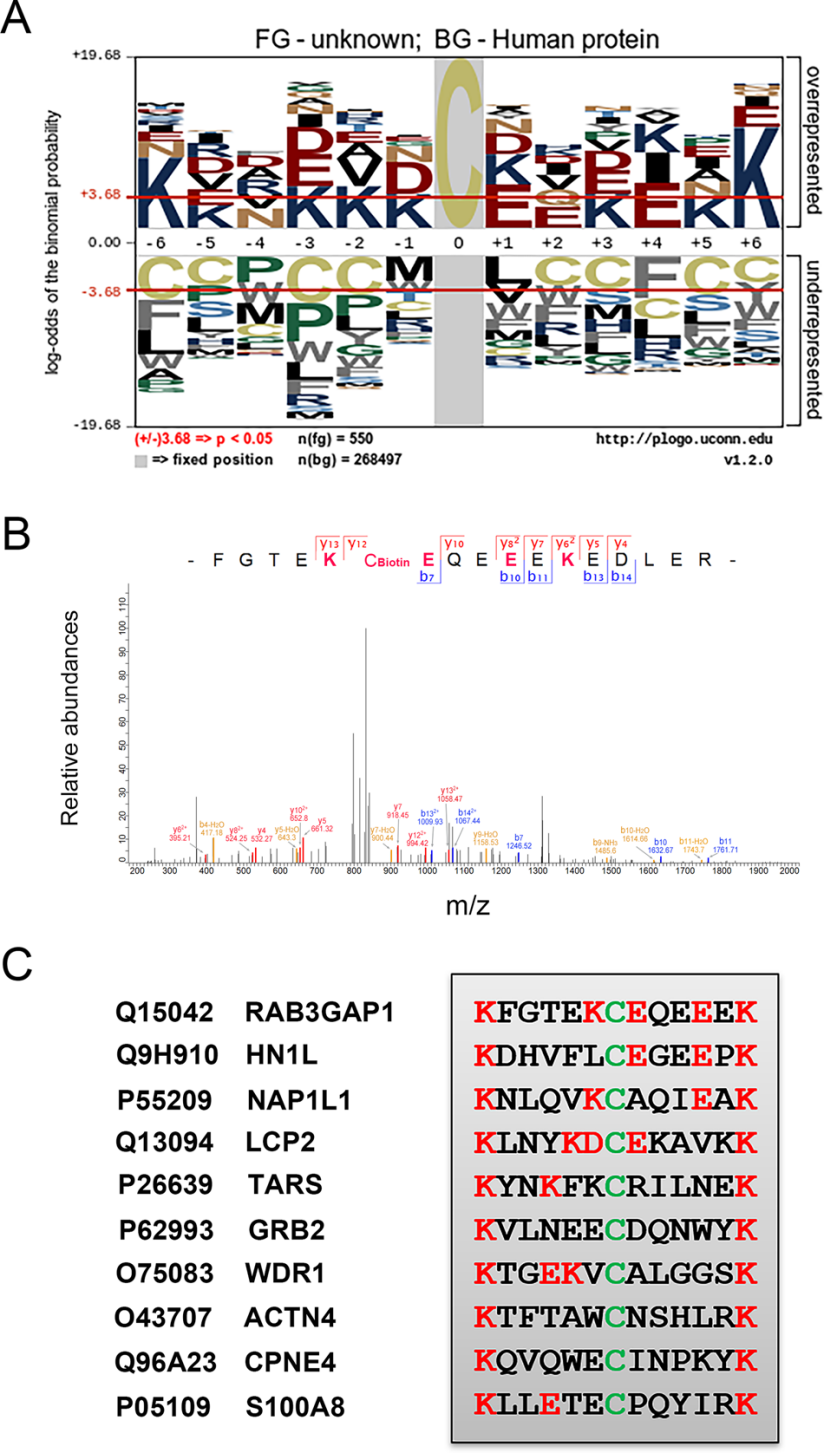
Consensus sequences of *S-*nitrosylated peptides in PDAC. **a** Consensus sequences derived from analysis of 592 *S-*nitrosylated peptides in pancreatic tissues and PANC-1 cells. **b** Mass spectrometric identification of Cys-858 in RAB3GAP1 (Rab3 GTPase-activating protein catalytic subunit 1) as *S-*nitrosylation site. **c** Representative *S-*nitrosylated peptides containing acid-base motif in pancreatic tissues and PANC-1 cells. Protein accession IDs and names were given on the left. *S-*nitrosylation residues were shown in green, and flaking residues matching the acid-base motif were shown in red. Detailed annotations of these proteins were shown in Supplemental Tables S3 and S6.

Previous evidences suggested that the amino acid composition flanking cysteine residues exert great impacts on the susceptibility and specificity of cysteines for redox-based *S-*nitrosylation^14,17^. To provide insights into driving determinants of protein *S-*nitrosylation, the composition features of amino acids in the proximity of 592 *S-*nitrosylated residues from pancreatic tissues and PANC-1 cells were analyzed using the pLogo approach^39^. We found that amino acid residues with basic and acidic side-chains were significantly overrepresented in the proximity of *S-*nitrosylated cysteine residues identified in pancreatic tissues and PANC-1 cells, including lysine (K) and glutamic acid (E) (Fig. 3a; Supplemental Tables S3 and S6).

Specifically, lysine was found to be significantly overrepresented in six residues among all 12 positions flanking SNO sites, including positions +6, −6, −2, −3, +3 and −1 (Fig. 3a). Also, glutamic acid at positions +4, +1 and −3, and Aspartic acid at position -1, were preferentially present in the flanking residues of *S-*nitrosylated cysteine sites identified in this study (Fig. 3a). Moreover, we showed that the frequency of lysine in the flanking residues was significantly higher than glutamic and aspartic acids (Fig. 3a and Supplemental Tables S3 and S6). For instance, the *S-*nitrosylated Cys-858 in RAB3GAP1 (Rab3 GTPase-activating protein catalytic subunit 1) is flanked by two lysine residues at positions +6 and −1, and several glutamic acid residues at proximal positions as well (Fig. 3b). Similarly, a number of other *S-*nitrosylated peptides identified in pancreatic tissues and PANC-1 cells were also featured by existence of multiple K or E residues flanking the modification sites, especially K at position +6 and −6 (Fig. 3c; Supplemental Tables S3 and S6). These results further supported the potential roles of acid-base motif in promoting redox modifications such as *S-*nitrosylation in pancreatic pathology.

### Functional categorization of *S-*nitrosylated proteins in PDAC

**Fig. 4:**
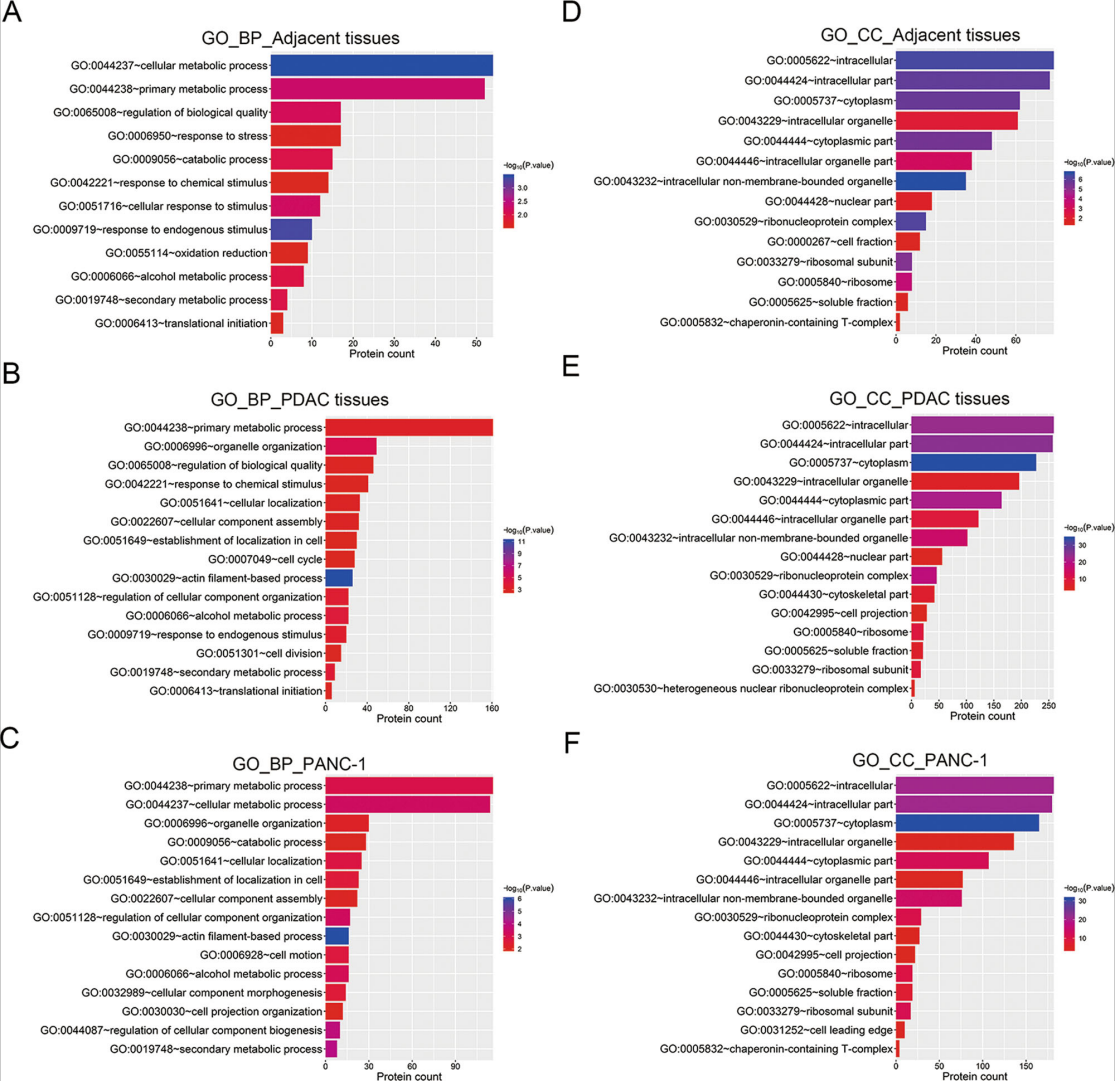
Functional categorization of *S-*nitrosylated proteins in PDAC. **a**–**c** Functional annotation of *S-*nitrosylated proteins by biological processes. Fifteen GO biological processes with highest enrichment (*P* < 0.05) of *S-*nitrosylated proteins identified in adjacent (**a**), PDAC tissues (**b**) and PANC-1 cells (**c**) were shown in bar charts. **d**–**f** Functional annotation of *S-*nitrosylated proteins by subcellular distribution. Fifteen GO cellular components with the highest enrichment (P < 0.05) of *S-*nitrosylated proteins identified in adjacent (**a**), PDAC tissues (**b**) and PANC-1 cells (**c**) were shown in bar charts. GO gene ontology, BP biological processes, CC cellular components.

To probe the biological roles of *S-*nitrosylation in PDAC, we performed a functional classification of these *S-*nitrosylated proteins identified in pancreatic tissues and cells through Gene Ontology (GO) analysis. *S-*nitrosylated proteins in adjacent pancreatic tissues, PDAC tissues and PANC-1 cells were separately analyzed (Fig. 4a–f). In adjacent non-cancerous tissues, *S-*nitrosylated proteins were mainly associated with basic biological processes such as primary cellular metabolism, regulation of biological quality, responses to stress and stimulus, catabolic processes, oxidation and reduction, secondary metabolism, and translational initiation (Fig. 4a). But in PDAC tissues and PANC-1 cells, *S-*nitrosylated proteins were specifically enriched in cell cycle, cell division, cell motion, and actin filament-based processes, which are all associated with tumorigenesis (Fig. 4b, c). Through GO annotation based on cellular components, we showed that *S-*nitrosylated proteins of these three groups covers various subcellular compartments, such as cytoplasm, nuclear parts, ribonucleoprotein complex, and ribosome (Fig. 4d–f), suggesting the widespread roles of protein *S-*nitrosylation in PDAC pathogenesis.

### KEGG pathways with enrichment of *S-*nitrosylated proteins in PDAC

**Fig. 5:**
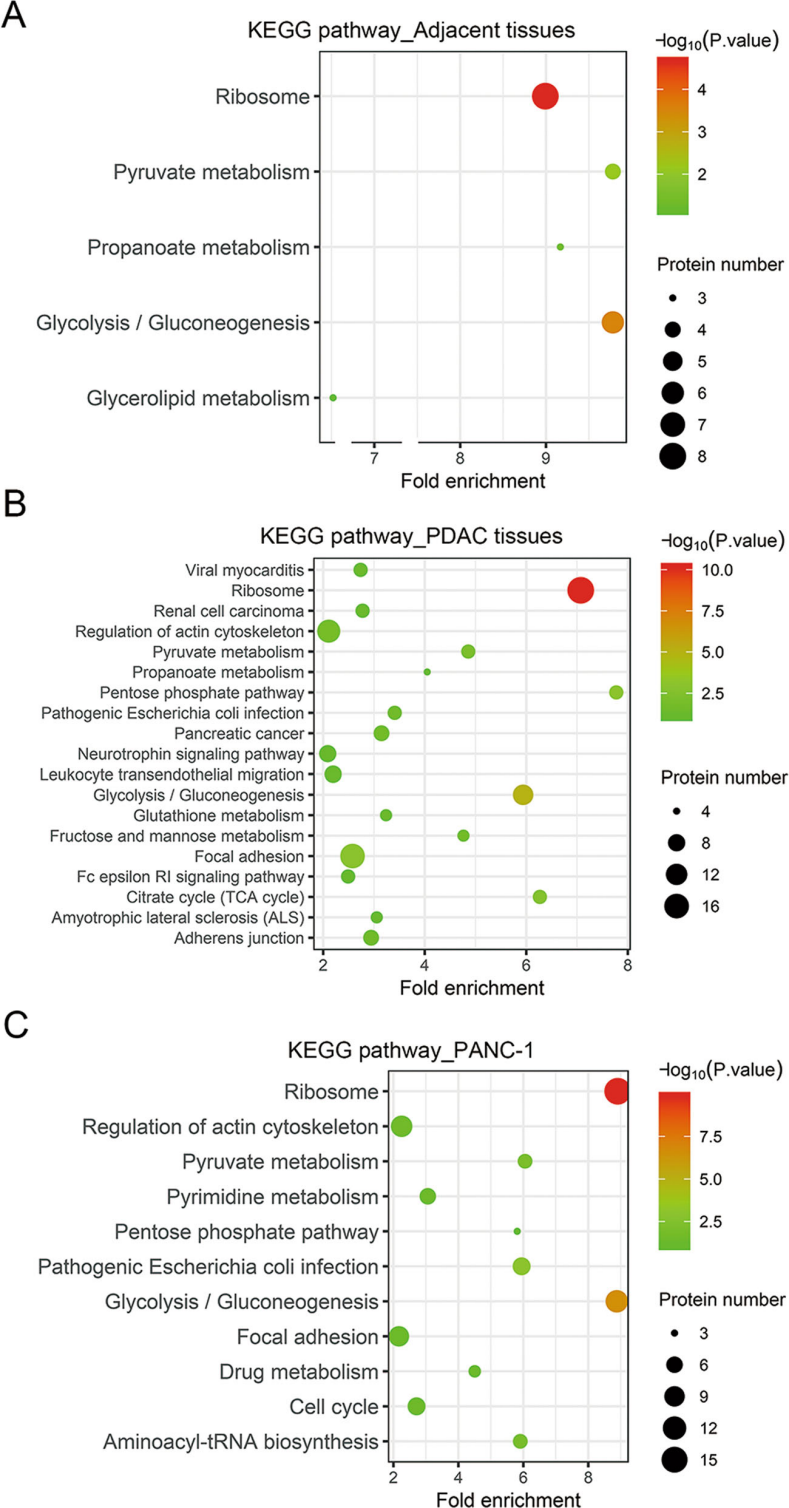
KEGG pathways of *S-*nitrosylated proteins identified in PDAC. The KEGG pathways with significant enrichment of *S-*nitrosylated proteins identified in adjacent (**a**), PDAC tissues (**b**) and PANC-1 cells (**c**) were obtained using the Database for Annotation, Visualization and Integrated Discovery (DAVID).

For more insights into the biological functions of protein *S-*nitrosylation in PADC pathogenesis, we further analyzed KEGG pathways with significant enrichment of *S-*nitrosylated proteins identified in our site-specific proteomics. In adjacent tissues, *S-*nitrosylated proteins were significantly linked with ribosome, pyruvate and propanoate metabolism, glycolysis and gluconeogenesis and glycerolipid metabolism (Fig. 5a). Apart from these metabolism processes, *S-*nitrosylated proteins in PDAC tissues and PANC-1 cells were significantly enriched in multiple biological processes associated with cancer initiation, development and metastasis, including the renal cell carcinoma, actin cytoskeleton regulation, pancreatic cancer, neurotrophin signaling, leukocyte trans-endothelial migration, focal adhesion, adherent junctions, and cell cycle pathways (Figs. 5b, c; Supplemental Fig. S4–S9). Significant enrichment of proteins in key pathways linked with cancer development further indicated the crucial roles of protein *S-*nitrosylation in PDAC pathogenesis.

### Enrichment of *S-*nitrosylated proteins in pancreatic cancer pathway

**Fig. 6:**
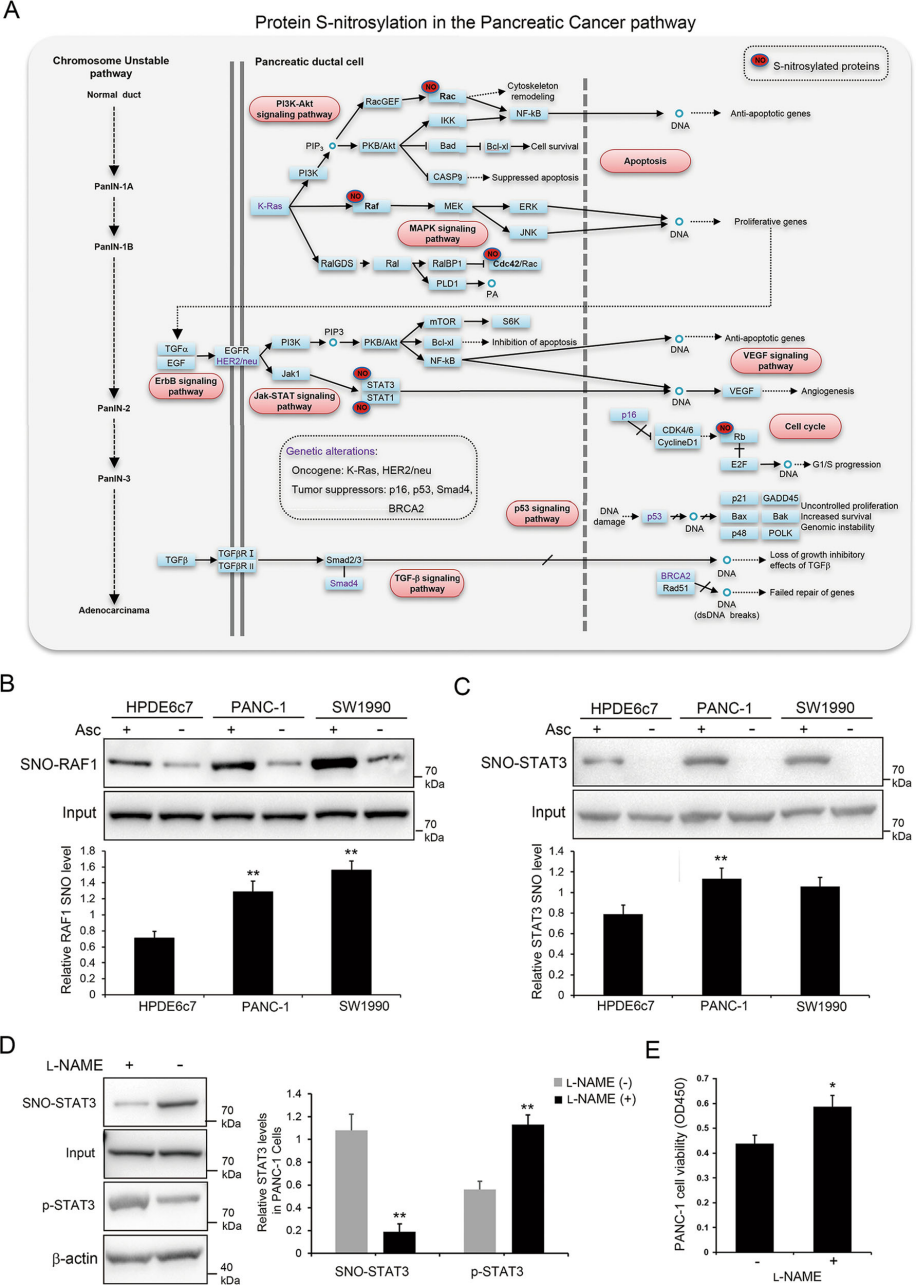
Extensive *S-*nitrosylation of signaling components underlying PDAC pathogenesis. **a** A schematic illustration of protein *S-*nitrosylation in pancreatic cancer pathway. *S-*nitrosylated proteins were significantly enriched in the pancreatic cancer pathway by KEGG pathway analysis. Pathway diagram was modified from KEGG pathway database (www.kegg.jp). **b, c** *S-*nitrosylation of Raf-1 and STAT3 proteins in HPDE6c7, PANC-1 and SW1990 cell lines. The biotin-switch assay was performed to confirm the endogenous *S-*nitrosylation of Raf-1 and STAT3 proteins. **d** *S-*nitrosylation and phosphorylation (Tyr^507^) of STAT3 proteins in PANC-1 cells treated with NOS inhibitor L-NAME. Protein S-nitrosylation were determined by biotin-switch method and phosphorylation was analyzed by western blot in cells treated with L-NAME (10 μM) for 48 h. **e** The viabilities of PANC-1 cells treated with _L_-NAME. PANC-1 cells were treated with _L_-NAME treatment for 48 h, followed by cell viability determination with CCK-8 method. L-NAME L-NG-nitroarginine methyl ester, Asc sodium ascorbate, SNO *S*-nitrosylated protein, Raf-1 v-raf-1 murine leukemia viral oncogene homolog 1, STAT3 signal transducer and activator of transcription 3, p-STAT3 phosphorylated STAT3, *P < 0.05; **P < 0.01.

Interestingly, our KEGG pathway analysis showed that a number of *S-*nitrosylated proteins identified in PDAC tissues and PANC-1 cells were well-known players in the pancreatic cancer pathway (Figs. 5b and 6a). These proteins include Ras*-*related C3 botulinum toxin substrate 1 (Rac1), Rac2, v-raf-1 murine leukemia viral oncogene homolog 1 (Raf-1), cell division cycle 42 (CDC42), Signal transducer and activator of transcription 1 (STAT1), STAT3 and retinoblastoma (RB) proteins, which are key regulators of cell apoptosis, cell cycle and other biological processes (Fig. 6a; Supplemental Tables S3 and S6). In addition, we demonstrated that a myriad of *S-*nitrosylated proteins identified in PDAC tissues and cells were closely associated with other cancer-related pathways. These proteins includes PCNA, DNA-dependent protein kinase (DNA-PK), minichromosome maintenance complex component 3/5 (MCM3/5), growth factor receptor-binding protein-2 (GRB2), Rho-associated coiled-coil kinase 2 (ROCK2), P21-activated kinase 2 (PAK2), Ras homolog gene family member A (RhoA), mitogen-activated protein kinase P38, vasodilator-stimulated phosphoprotein (VASP) and tyrosine 3-monooxygenase/tryptophan 5-monooxygenase activation protein (14-3-3), all of which are widely involved in cell cycle, focal adhesion, adherent junction and cytoskeleton regulations (Supplemental Tables S3 and S6; Supplemental Figs. S4–S9).

### Suppression of PDAC cell viability by STAT3 protein *S*-nitrosylation

To validate protein *S-*nitrosylation in pancreatic cancer pathway, a biotin-switch method was performed to test the *S-*nitrosylation of Raf-1 and STAT3 proteins in non-neoplastic human pancreatic ductal epithelium cell line HPDE6c7 and two PDAC cell lines PANC-1 and SW1990. We showed that both Raf-1 and STAT3 proteins were highly *S-*nitrosylated in PANC-1 and SW1990 cells, compared with HPDE6c7 cells (Fig. 6b, c). Moreover, NOS inhibitor _L_-NAME treatment significantly decreased total protein S-nitrosylation level and suppressed STAT3 protein *S*-nitrosylation in PANC-1 cells (Fig. 6d; Supplemental Fig. S3), which resulted into increased STAT3 protein phosphorylation and PANC-1 cell viability (Fig. 6d, e), which further indicated the pathogenic roles of *S*-nitrosylation of key signaling components such as STAT3 in tumorigenesis. Taken together, these results suggest that signaling pathways promoting PDAC initiation and progression are subjected to extensive post-translational regulation by protein *S-*nitrosylation. The pathogenic roles of these *S-*nitrosylated proteins in pancreatic cancer cells deserve further investigation.

## Discussion

Accumulating evidences have established *S-*nitrosylation, a NO-induced protein redox modification, as one major mechanism underlying cancer initiation and development^6,17–19^, but its roles in PDAC pathogenesis still remains largely unexplored. Proteomics was proven capable of characterizing large number of novel *S-*nitrosylated proteins, which brought about substantial breakthroughs in functional investigations^30,32,34,35^. In the present study, we performed a site-specific proteomic analysis of endogenously *S-*nitrosylated proteins in pancreatic tissues surgically collected from PDAC patients, as well as the widely applied PDAC cell line PANC-1. The combined proteomic assays identified nearly 585 unique *S-*nitrosylation sites among 434 proteins, suggesting critical roles of extensive protein *S-*nitrosylation in PDAC pathogenesis. The *S-*nitrosylation of over one third of these proteins were also identified in previous reports, which validated the reliability of our protein *S-*nitrosylation dataset. It is worth mentioning that investigations using other cell lines with distinct origins might provide further illumination. Finally, we showed that NOS inhibitor effectively repressed STAT3 *S*-nitrosylation in PANC-1 cells, which induced great alteration of STAT3 phosphorylation and PANC-1 cell viability, indicative of the regulatory roles of protein *S*-nitrosylation in pancreatic cancer development. Further functional investigations would broaden our understanding of NO and protein *S*-nitrosylation in PDAC pathogenesis, also providing new candidates for targeted cancer therapies.

Protein *S*-nitrosylation and other redox modifications possesse essential roles in tumorigenesis and high potential for anti-cancer drug discovery^17,42,43^. The larger number of *S-*nitrosylated proteins were identified in PDAC tissues and PANC-1 cells, compared with the adjacent tissues, which is consistent with previously revealed NO overproduction associated with PDAC development^8–11,44,45^. These proteins specifically *S-*nitrosylated in context of PDAC are attractive targets for cancer biology research. In the past decade, extensive efforts were made to elucidate the chemical properties facilitating protein *S-*nitrosylation formation^14,17^. Our consensus sequence analysis revealed significant presence of lysine residues flanking *S-*nitrosylated cysteines in pancreatic cancer, further supporting the acid-base motif in facilitating protein *S-*nitrosylation as proposed in previous reports^32,34^. The *S*-nitrosylation residues identified by our site-specific proteomics would greatly accelerate further functional investigations in PDAC.

Our functional categorization showed the association of *S-*nitrosylated proteins with various biological processes in pancreatic physiology and PDAC pathogenesis, such as cell cycle, focal adhesion, adherent junction, and cytoskeleton regulations. More importantly, *S-*nitrosylated proteins identified from PDAC tissues and PANC-1 cells were significantly enriched in the pancreatic cancer pathway, as shown by KEGG annotation. For instance, Raf-1 acts as a key player promoting activation MEK-MAPK pathway in PDAC cells^46^, and the *S-*nitrosylation of Raf-1 identified in our proteomic assay provided new clue for study of Raf-1 activity modulation in PDAC pathogenesis. Moreover, STAT3 activation via phosphorylation exerts crucial roles in pancreatic cancer development^47,48^. Interestingly, previous studies also demonstrated that STAT3 *S-*nitrosylation suppressed microglia proliferation, multiple myeloma cell survival and proliferation microglia proliferation, multiple myeloma cell proliferation and abnormal proliferation by inhibiting STAT3 phosphorylation^49–51^. In this study, STAT3 was also identified as *S*-nitrosylated protein in the context of pancreatic cancer, and NOS inhibitor caused decreased STAT3 *S*-nitrosylation and elevated STAT3 phosphorylation and pancreatic cancer cell viability, which is consistent with previous reports of STAT3 *S*-nitrosylation in other diseases^49–51^ and highlighted the roles of STAT3 *S*-nitrosylation in tumorigenesis. *S-*nitrosylated proteins in pancreatic cancer pathway identified in this study also include Rac1, Rac2, CDC42, STAT1, and RB, which are all important regulators of pancreatic cancer cells.

In addition, cellular metabolism reprogramming serves as a critical mechanism for cancer cells to maintain viability and build new biomass^52,53^. For instance, abnormal glucose and amino acid metabolism plays essential roles in the etiology and mortality of pancreatic cancer^54,55^. In this study, we also identified several key enzymes in glycolysis, gluconeogenesis, and phenylalanine metabolism, such as phosphoglycerate kinase 1 (PGK1), triosephosphate isomerase (TPI1) and aldehyde dehydrogenase family 1, member A3 (ALDH1A3), indicative of the regulation of cancer cell metabolism by *S-*nitrosylation during PDAC development. Proteins in other cancer-related processes, including pyruvate and propanoate metabolism, glycerolipid metabolism, cell cycle progression, focal adhesion, adherent junctions, neurotrophin signaling, and leukocyte trans-endothelial migration, were extensively *S-*nitrosylated as well. Large number of target proteins suggested the pleiotropic effects of protein *S-*nitrosylation on PDAC pathogenesis, which deserve further investigation.

Taking together, our site-specific proteomics identified a large number of endogenously *S-*nitrosylated proteins and their modification sites in PDAC tissues and cells, involving multiple processes and signaling pathways closely associated with pancreatic tumorigenesis. NOS inhibitor treatment significantly suppressed STAT3 *S*-nitrosylation, promoted STAT3 phosphorylation and enhanced viability of pancreatic cancer cells, further indicating the essential roles of *S*-nitrosylation in PDAC pathogenesis. These findings provided novel insights into nitric oxide signaling and *S*-nitrosylation in pancreatic cancer development, and also a basis for protein modification-based cancer diagnosis and treatment.

## Supplementary information

Supplemental Figure Legends

Supplemental Figure S1

Supplemental Figure S2

Supplemental Figure S3

Supplemental Figure S4

Supplemental Figure S5

Supplemental Figure S6

Supplemental Figure S7

Supplemental Figure S8

Supplemental Figure S9

Supplemental Table S1

Supplemental Table S2

Supplemental Table S3

Supplemental Table S4

Supplemental Table S5

Supplemental Table S6

Supplemental Table S7

Supplemental Table S8
