# Supplementary material for: Extensive protein S-nitrosylation associated with human pancreatic ductal adenocarcinoma pathogenesis
Source: Cell Death Dis. 2019 Dec 4;10(12):914. doi: 10.1038/s41419-019-2144-6 (PMC6892852; doi:10.1038/s41419-019-2144-6)
Supplement: Supplementary file 1 — Supplemental Figure Legends [file 41419_2019_2144_MOESM1_ESM.docx]

**Supplementary Figures:**

**Supplemental Figure S1. Increased NOS expression in cancerous tissues from PDAC patients.**

Protein abundances of three NO synthases iNOS, eNOS and nNOS between PDAC and adjacent non-cancerous tissues were analyzed by western blotting. GAPDH was used as the internal standard.

**Supplemental Figure S2. Total protein *S-*nitrosylation levels in PDAC cancerous and adjacent tissues.**

Total levels of protein *S-*nitrosylation between PDAC and adjacent non-cancerous tissues were determined by biotin-switch method. Pancreatic tissue lysates without Asc treatment during biotin-switch assay were used as negative control. PDAC: pancreatic ductal adenocarcinoma; Asc: sodium ascorbate; SNO: *S-*nitrosylated protein; *P < 0.05.

**Supplemental Figure S3. Decreased protein S-nitrosylation in PANC-1 cells viability induced by _L_-NAME treatment.**

Total protein S-nitrosylated protein levels in PANC-1 cells treated with NOS inhibitor _L_-NAME. Protein S-nitrosylation levels were determined by biotin-switch method after _L_-NAME treatment (10 μM) for 48 h. _L_-NAME: L-NG-Nitroarginine Methyl Ester; Asc: sodium ascorbate; SNO: *S-*nitrosylated protein;

**Supplemental Figure S4. *S-*nitrosylated proteins in the cell cycle pathway.**

A schematic illustration of *S-*nitrosylated proteins significantly enriched in the cell cycle pathway modified from KEGG pathway database ([www.kegg.jp](http://www.kegg.jp)).

**Supplemental Figure S5. *S-*nitrosylated proteins in the** **focal adhesion pathway.**

A schematic illustration of *S-*nitrosylated proteins significantly enriched in the focal adhesion pathway modified from KEGG pathway database ([www.kegg.jp](http://www.kegg.jp)).

**Supplemental Figure S6. *S-*nitrosylated proteins in the adherent junction pathway.**

A schematic illustration of *S-*nitrosylated proteins significantly enriched in the adherent junction pathway modified from KEGG pathway database ([www.kegg.jp](http://www.kegg.jp)).

**Supplemental Figure S7. *S-*nitrosylated proteins in the** **leukocyte tran*s-*endothelial migration pathway.**

A schematic illustration of *S-*nitrosylated proteins significantly enriched in the leukocyte tran*s-*endothelial migration pathway modified from KEGG pathway database ([www.kegg.jp](http://www.kegg.jp)).

**Supplemental Figure S8. *S-*nitrosylated proteins in the** **neurotrophin signaling pathway.**

A schematic illustration of *S-*nitrosylated proteins significantly enriched in the neurotrophin signaling modified from KEGG pathway database ([www.kegg.jp](http://www.kegg.jp)).

**Supplemental Figure S9. *S-*nitrosylated proteins in regulation of actin cytoskeleton.**

A schematic illustration of *S-*nitrosylated proteins significantly enriched in the actin cytoskeleton regulation modified from KEGG pathway database ([www.kegg.jp](http://www.kegg.jp)).

**Supplemental Tables:**

**Supplemental Table S1. Clinical data of PDAC patients for tissue collection.**

Clinical data of four PDAC patients, from which PDAC tissues and paired adjacent non-cancerous pancreatic tissues were collected including sexuality, age of diagnosis, histology diagnosis, differentiation degree, metastasis status and TNM (tumor-node-metastasis) staging (8^th^ edition) proposed by the American Joint Committee on Cancer (AJCC).

**Supplemental Table S2. Total numbers of biotinylated peptides identified in pancreatic tissues and cells.**

Total numbers of biotinylated peptides identified in each replicate during proteomic analysis of adjacent non-cancerous tissues, PDAC tissues and PANC-1 cells were listed in this table, as well as the numbers of biotinylated peptides identified in negative controls and common peptides identified in all four biological replicates.

**Supplemental Table S3. A list of *S-*nitrosylated peptides and proteins in PDAC patients.**

Accessions, names, annotations, *S-*nitrosylation sites, PEP Score and peptide intensities of *S-*nitrosylated proteins identified in PDAC and adjacent tissues were listed. *S-*nitrosylated proteins also identified in PANC-1 cells were marked and the PMIDs of previous reports covering *S-*nitrosylated proteins in this study were provided as well.

**Supplemental Table S4. Detailed information of *S*-nitrosylated peptides identified in PDAC patients.**

Detailed mass spectrometry information of each *S*-nitrosylated peptide from PDAC patients were listed, including scan number, missed cleavages, modification probabilities, charges, mass error [ppm], score, localization probability and other indexes.

**Supplemental Table S5. A list of peptides with** **ambiguous modification site assignments in PDAC patients.**

Detailed mass spectrometry information of *S*-nitrosylated peptides from PDAC patients with ambiguous modification site assignments were listed, including scan number, missed cleavages, modification probabilities, charges, mass error [ppm], score, localization probability and other indexes.

**Supplemental Table S6. A list of *S-*nitrosylated peptides and proteins in PANC-1 cells.**

The accessions, names, annotations, *S-*nitrosylation sites, PEP Score and peptide intensities of *S-*nitrosylated proteins identified in PANC-1 cells were listed. *S-*nitrosylated proteins also identified in human pancreatic tissues were marked and the PMIDs of previous reports covering *S-*nitrosylated proteins in this study were provided as well.

**Supplemental Table S7. Detailed information of S-nitrosylated peptides identified in PANC-1 cells.**

Detailed mass spectrometry information of *S*-nitrosylated peptides from PANC-1 cells with ambiguous modification site assignments were listed, including scan number, missed cleavages, modification probabilities, charges, mass error [ppm], score, localization probability and other indexes.

**Supplemental Table S8. A list of peptides with ambiguous modification site assignments in PANC-1 cells.**

Detailed mass spectrometry information of *S*-nitrosylated peptides from PANC-1 cells with ambiguous modification site assignments were listed, including scan number, missed cleavages, modification probabilities, charges, mass error [ppm], score, localization probability and other indexes.
