# Supplementary figures and images for: Extensive protein S-nitrosylation associated with human pancreatic ductal adenocarcinoma pathogenesis
Source: Cell Death Dis. 2019 Dec 4;10(12):914. doi: 10.1038/s41419-019-2144-6 (PMC6892852; doi:10.1038/s41419-019-2144-6)

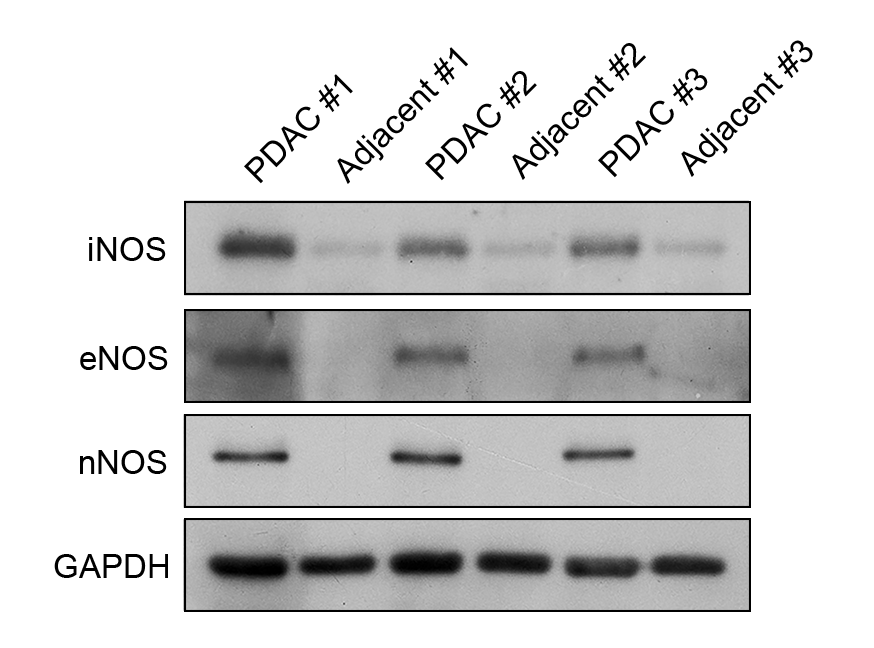

Supplement: Supplementary file 2 — Supplemental Figure S1 [file 41419_2019_2144_MOESM2_ESM.tif]

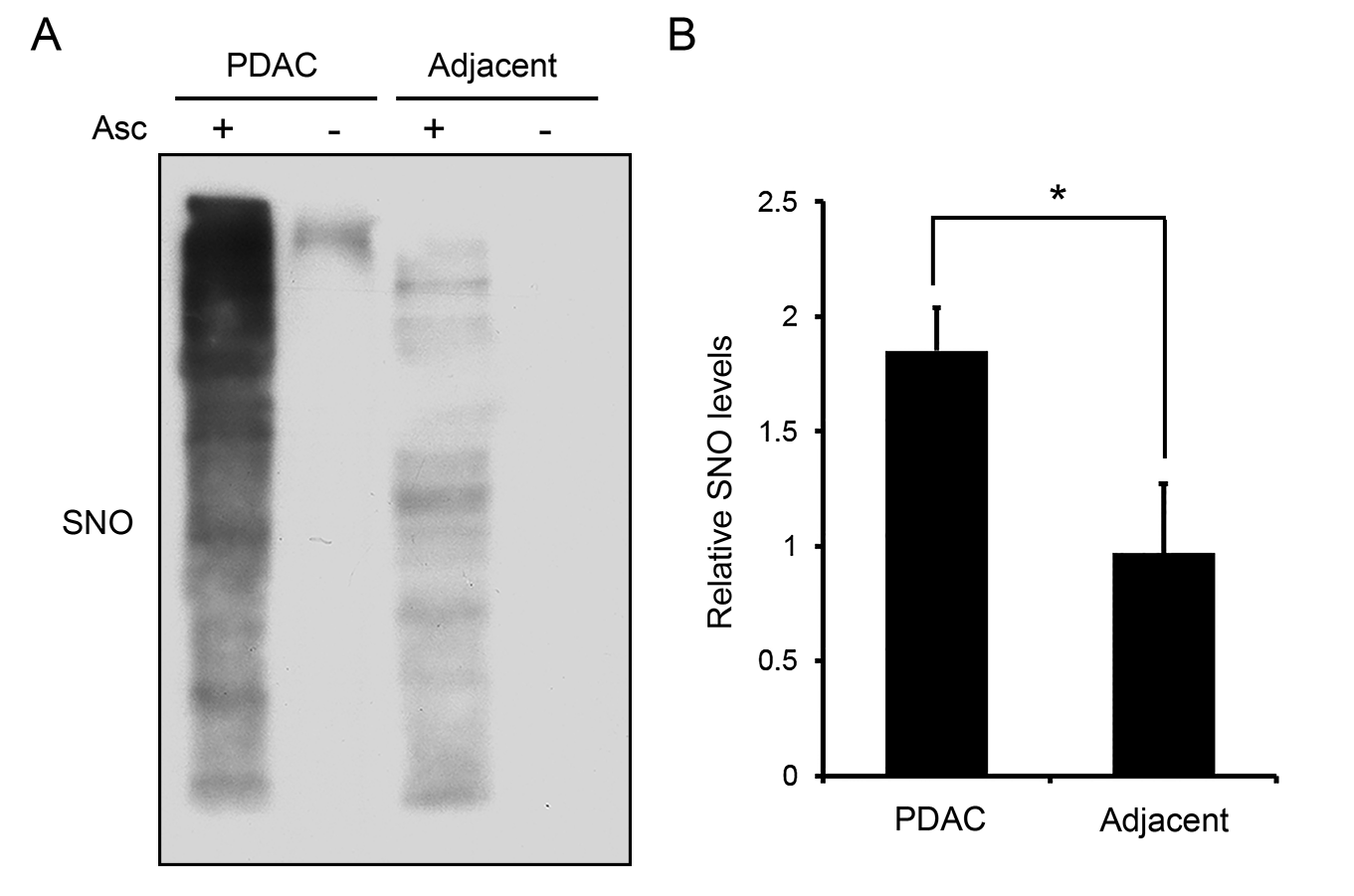

Supplement: Supplementary file 3 — Supplemental Figure S2 [file 41419_2019_2144_MOESM3_ESM.tif]

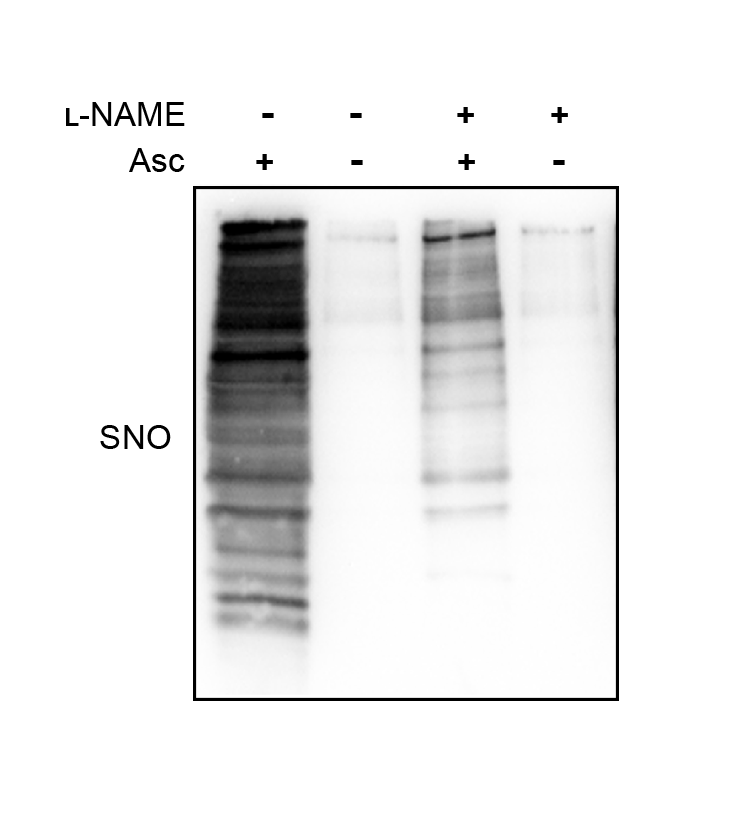

Supplement: Supplementary file 4 — Supplemental Figure S3 [file 41419_2019_2144_MOESM4_ESM.tif]

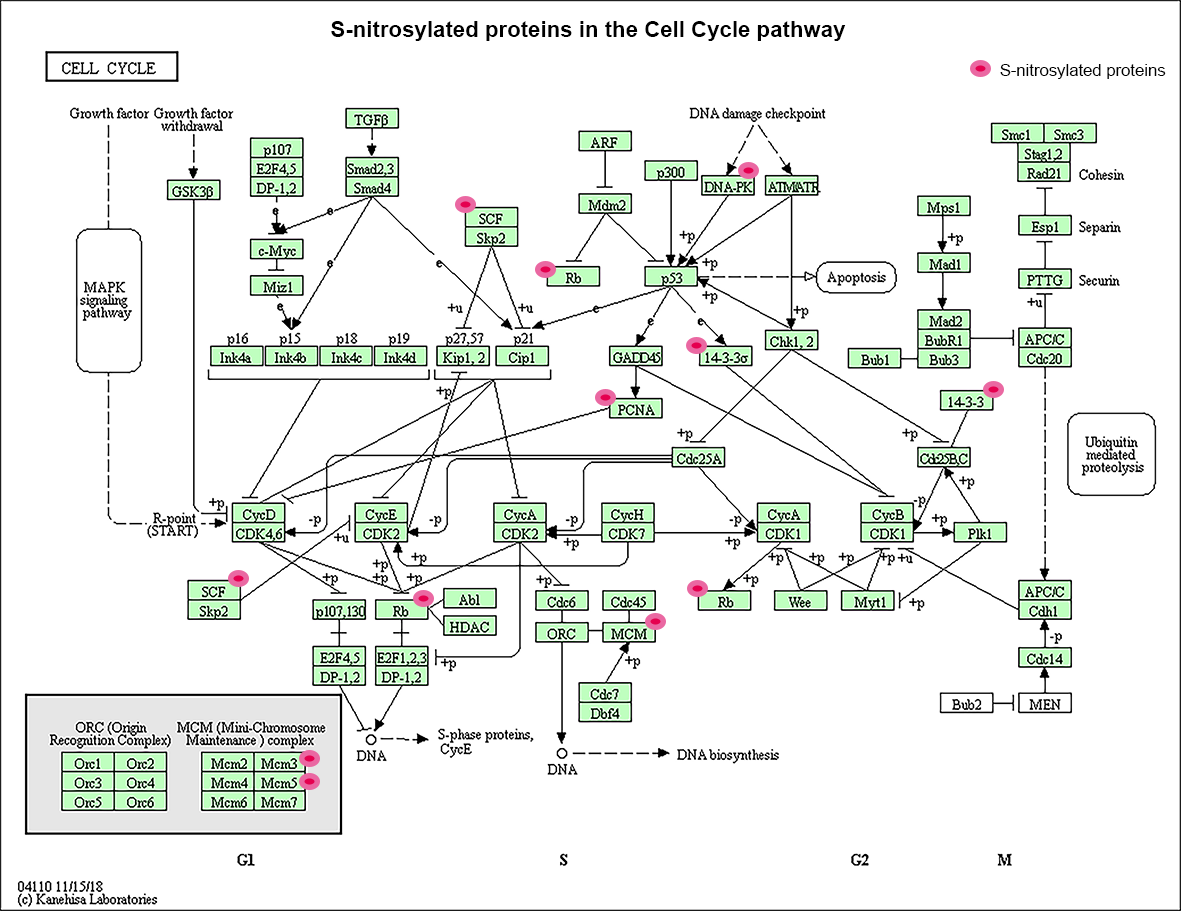

Supplement: Supplementary file 5 — Supplemental Figure S4 [file 41419_2019_2144_MOESM5_ESM.tif]

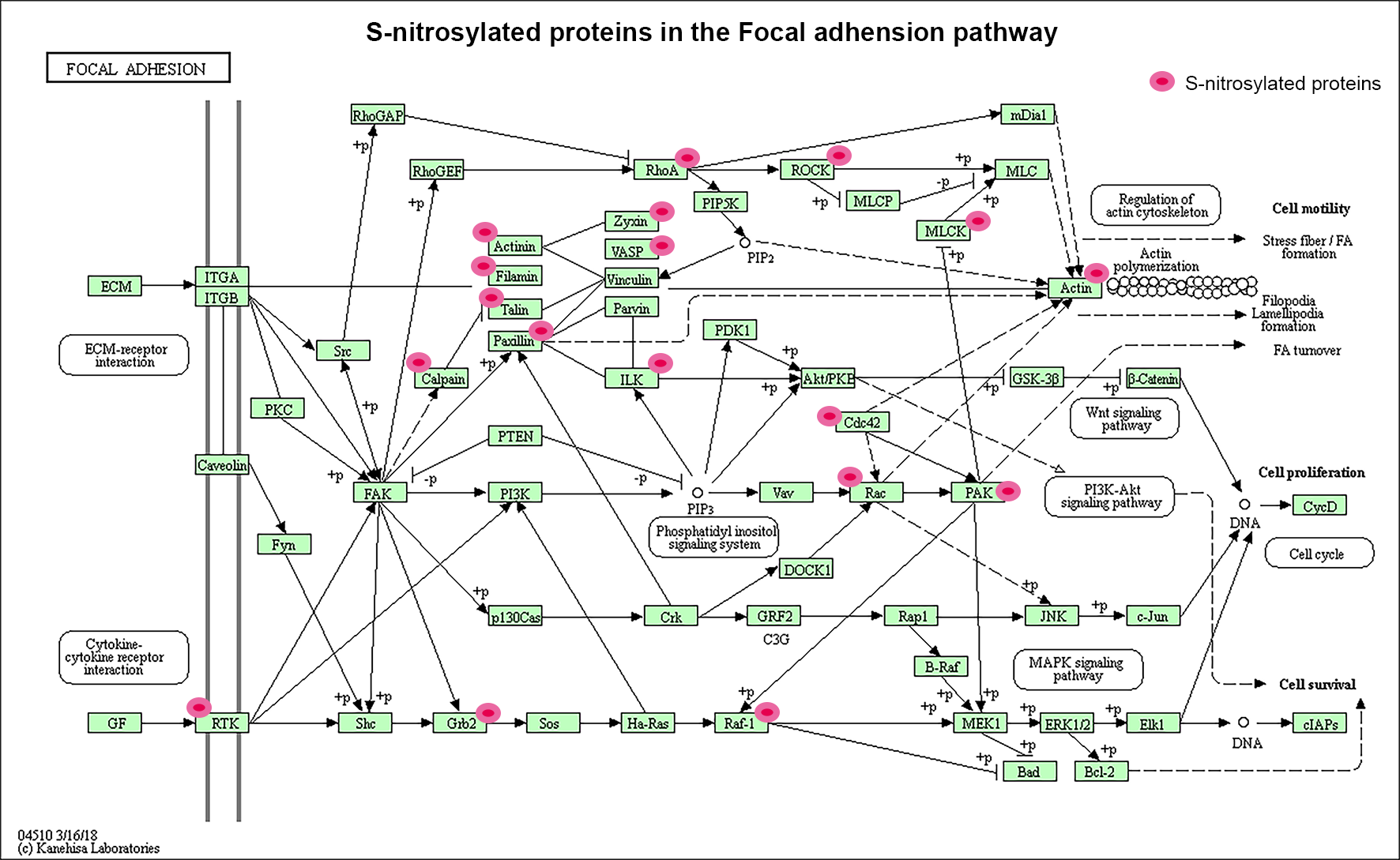

Supplement: Supplementary file 6 — Supplemental Figure S5 [file 41419_2019_2144_MOESM6_ESM.tif]

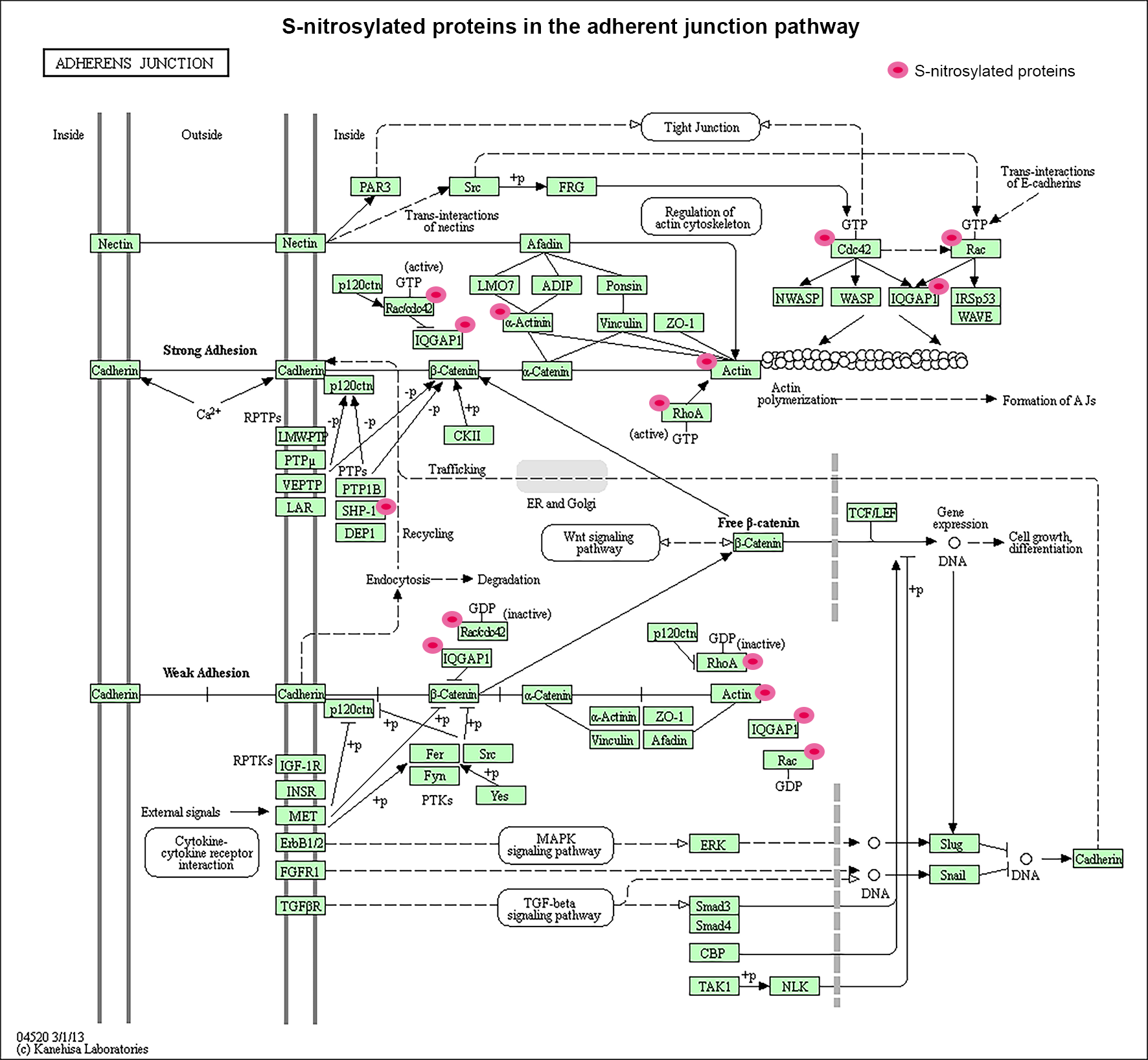

Supplement: Supplementary file 7 — Supplemental Figure S6 [file 41419_2019_2144_MOESM7_ESM.tif]

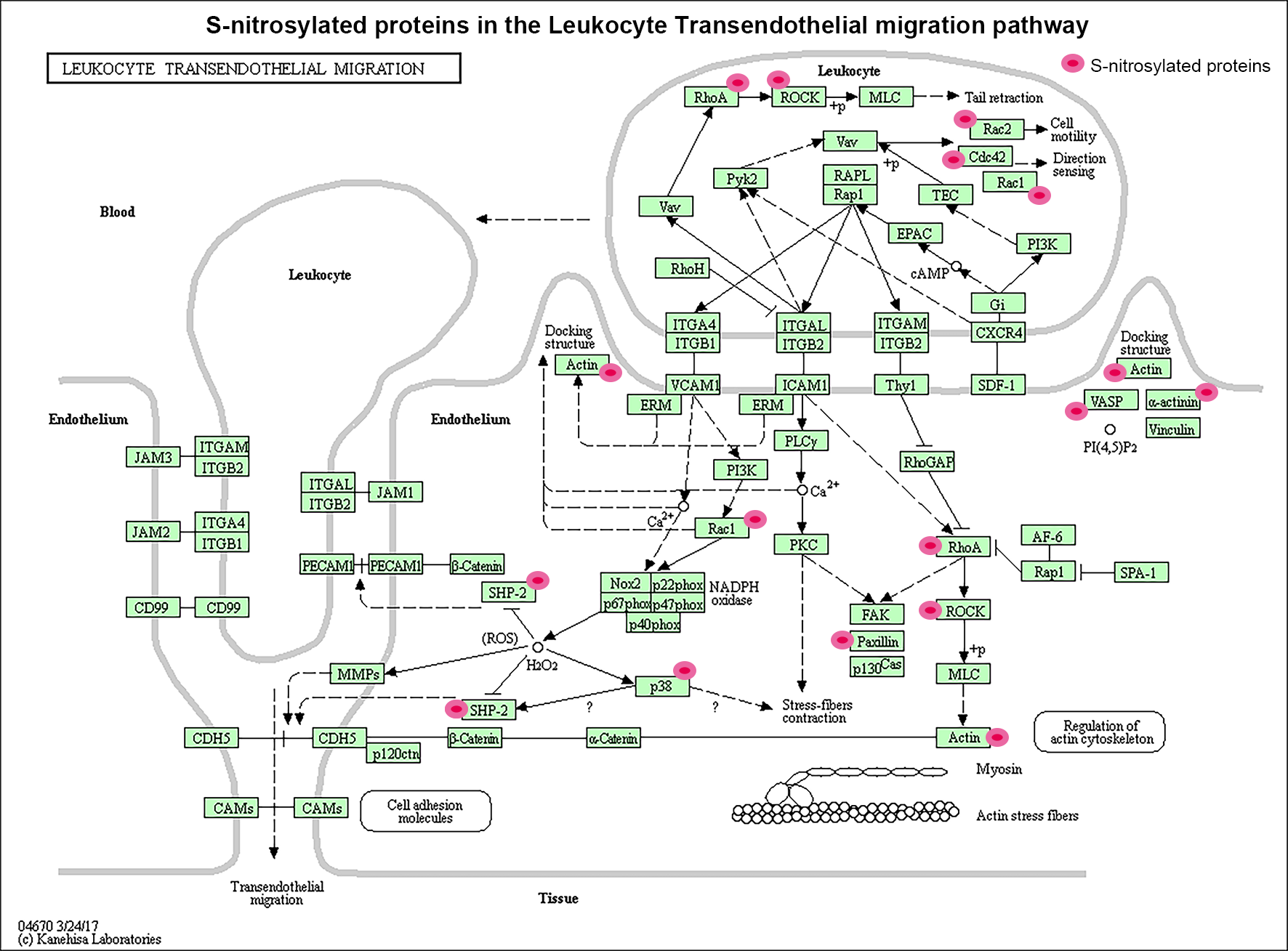

Supplement: Supplementary file 8 — Supplemental Figure S7 [file 41419_2019_2144_MOESM8_ESM.tif]

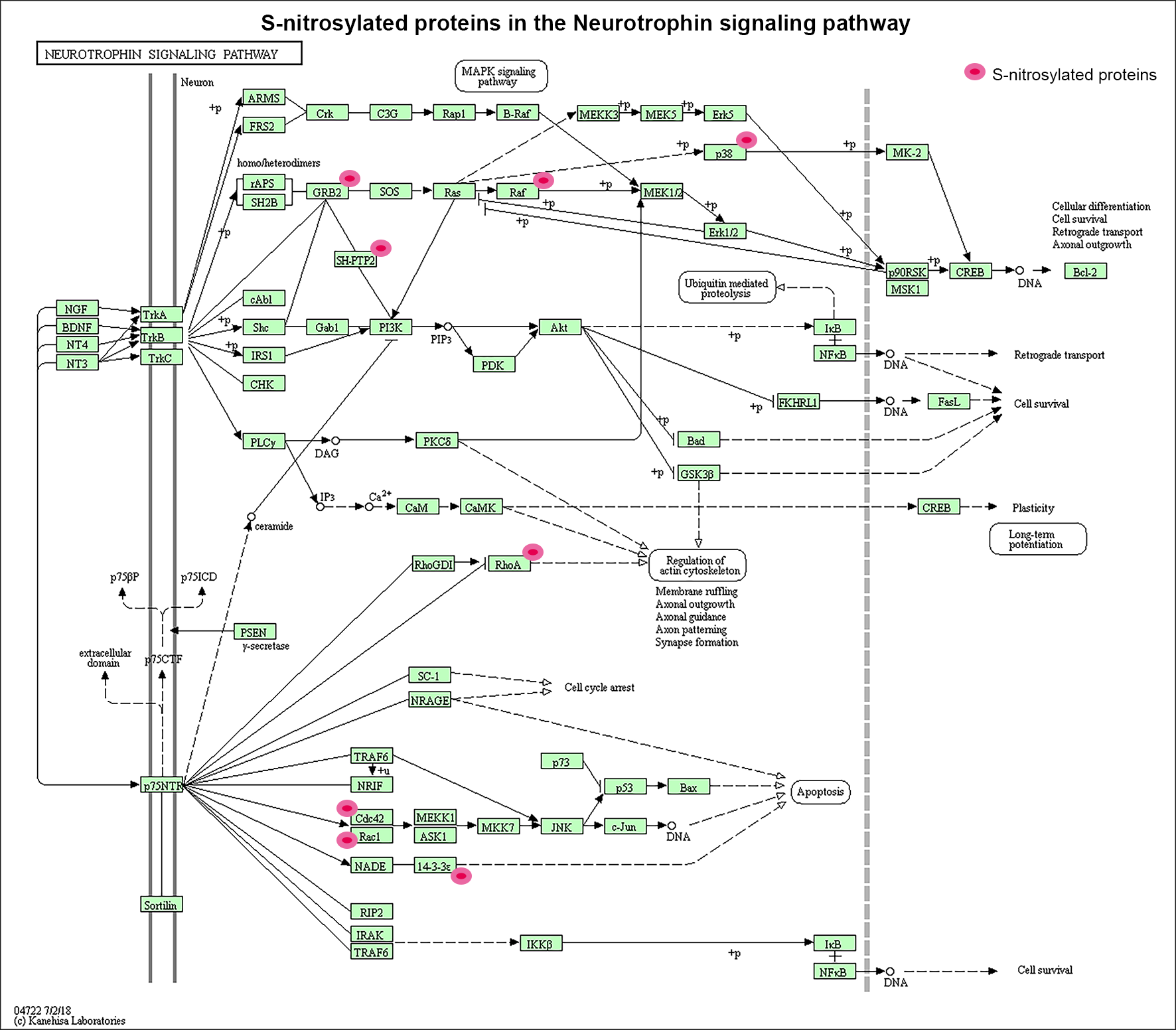

Supplement: Supplementary file 9 — Supplemental Figure S8 [file 41419_2019_2144_MOESM9_ESM.tif]

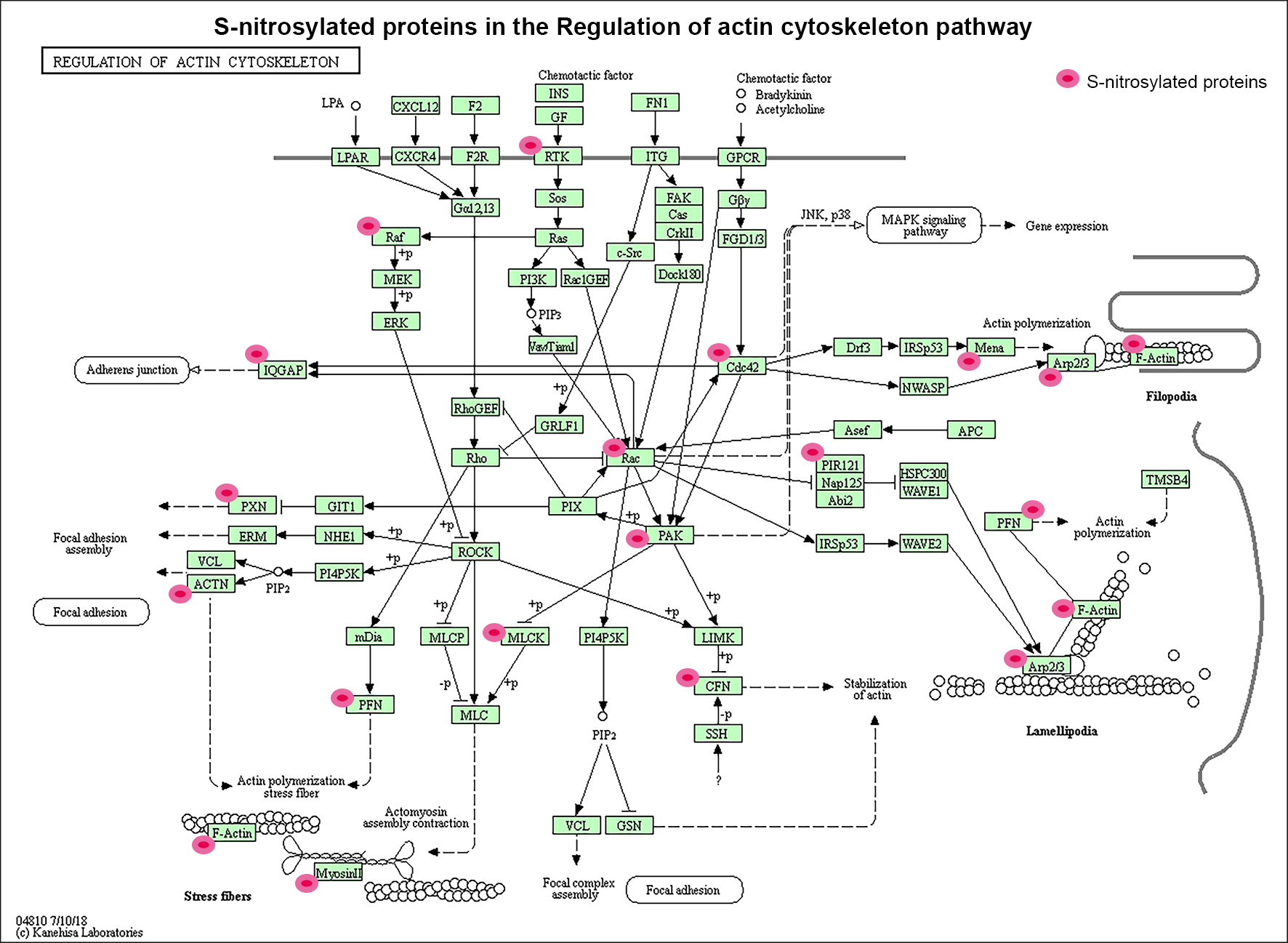

Supplement: Supplementary file 10 — Supplemental Figure S9 [file 41419_2019_2144_MOESM10_ESM.tif]

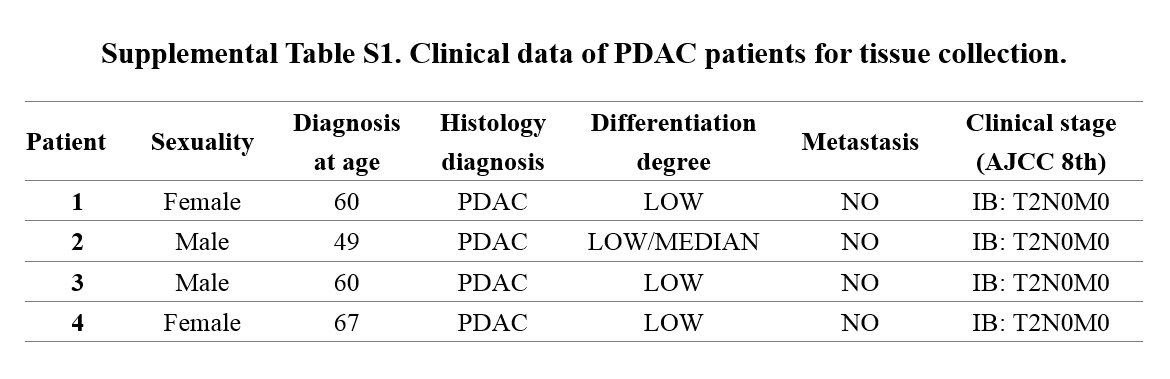

Supplement: Supplementary file 11 — Supplemental Table S1 [file 41419_2019_2144_MOESM11_ESM.tif]

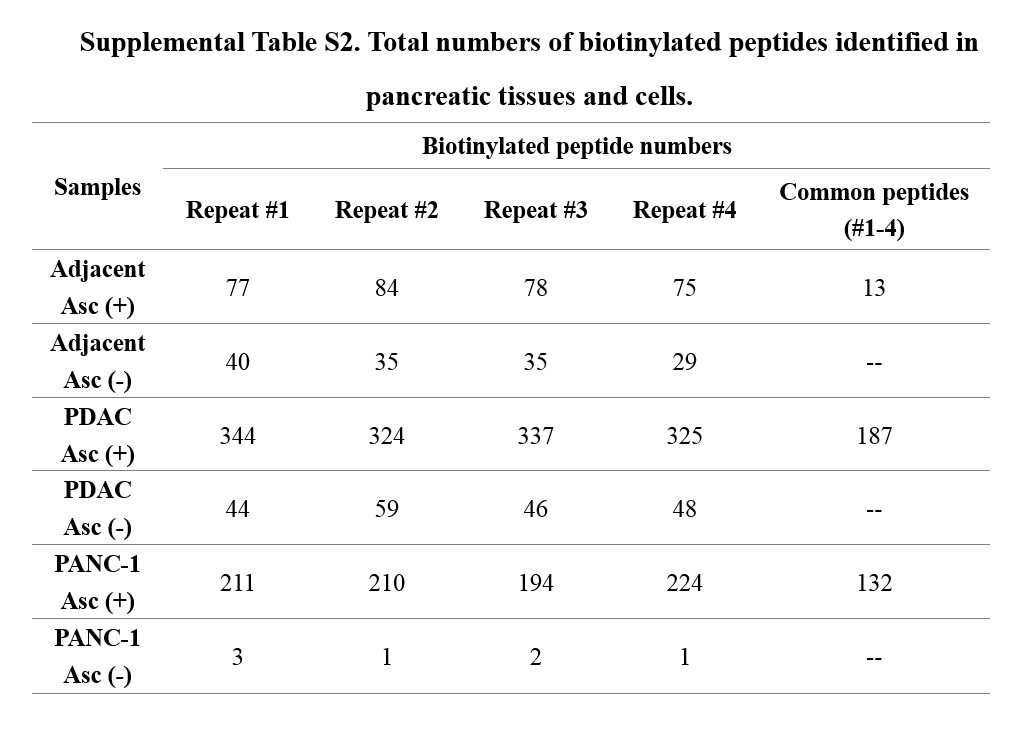

Supplement: Supplementary file 12 — Supplemental Table S2 [file 41419_2019_2144_MOESM12_ESM.tif]
